# T cell mediated immunity induced by the live-attenuated *Shigella flexneri* 2a vaccine candidate CVD 1208S in humans

**DOI:** 10.1186/s12967-018-1439-1

**Published:** 2018-03-13

**Authors:** Franklin R. Toapanta, Paula J. Bernal, Karen L. Kotloff, Myron M. Levine, Marcelo B. Sztein

**Affiliations:** 1 Department of Medicine, Center for Vaccine Development, University of Maryland School of Medicine, Baltimore, MD 21201 USA; 2 Department of Pediatrics, Center for Vaccine Development, University of Maryland School of Medicine, Baltimore, MD 21201 USA

**Keywords:** *Shigella flexneri* 2a, Oral vaccine, CVD 1208S, T cell mediated immunity, IpaB, Nanoparticles

## Abstract

**Background:**

Shigellosis persists as a public health problem worldwide causing ~ 165,000 deaths every year, of which ~ 55,000 are in children less than 5 years of age. No vaccine against shigellosis is currently licensed. The live-attenuated *Shigella flexneri* 2a vaccine candidate CVD 1208S (*S. flexneri 2a*; Δ*guaBA*, Δ*set*, Δ*sen*) demonstrated to be safe and immunogenic in phase 1 and 2 clinical trials. Earlier reports focused on humoral immunity. However, *Shigella* is an intracellular pathogen and therefore, T cell mediated immunity (T-CMI) is also expected to play an important role. T-CMI responses after CVD 1208S immunization are the focus of the current study.

**Methods:**

Consenting volunteers were immunized orally (3 doses, 10^8^ CFU/dose, 28 days apart) with CVD 1208S. T-CMI to IpaB was assessed using autologous EBV-transformed B-Lymphocytic cell lines as stimulator cells. T-CMI was assessed by the production of 4 cytokines (IFN-γ, IL-2, IL-17A and TNF-α) and/or expression of the degranulation marker CD107a in 14 volunteers (11 vaccine and 3 placebo recipients).

**Results:**

Following the first immunization, T-CMI was detected in CD8 and CD4 T cells obtained from CVD 1208S recipients. Among CD8 T cells, the T effector memory (T_EM_) and central memory (T_CM_) subsets were the main cytokine/CD107a producers/expressors. Multifunctional (MF) cells were also detected in CD8 T_EM_ cells. Cells with 2 and 3 functions were the most abundant. Interestingly, TNF-α appeared to be dominant in CD8 T_EM_ MF cells. In CD4 T cells, T_EM_ responses predominated. Following subsequent immunizations, no booster effect was detected. However, production of cytokines/expression of CD107a was detected in individuals who had previously not responded. After three doses, production of at least one cytokine/CD107a was detected in 8 vaccinees (73%) in CD8 T_EM_ cells and in 10 vaccinees (90%) in CD4 T_EM_ cells.

**Conclusions:**

CVD 1208S induces diverse T-CMI responses, which likely complement the humoral responses in protection from disease.

*Trial registration* This study was approved by the Institutional Review Board and registered on ClinicalTrials.gov (identifier NCT01531530)

**Electronic supplementary material:**

The online version of this article (10.1186/s12967-018-1439-1) contains supplementary material, which is available to authorized users.

## Background

*Shigella* is the enteroinvasive bacterium responsible for bacillary dysentery (shigellosis). *Shigella* causes ~ 165,000 deaths worldwide every year, of which ~ 55,000 are in children younger than 5 years of age [1, 2]. In recent years the treatment of shigellosis has become increasingly difficult as resistance to antibiotics has spread [3]. Therefore, new approaches to treat and/or prevent shigellosis are highly desirable. Vaccines have proven to be an effective method to prevent various infectious diseases. Human studies have shown that a previous *Shigella* infection confers up to 72% protection against subsequent disease episodes [4–7]; therefore an effective *Shigella* vaccine could significantly reduce the burden of this disease. However, to date, no vaccine has been licensed for *Shigella*. Various promising live-attenuated oral vaccine candidates are currently under development, such as the live-attenuated vaccine candidate CVD 1208S (*S. flexneri* 2a; *ΔguaBA, Δset, Δsen*) [8, 9]. Phases 1 and 2 clinical trials of this vaccine candidate have demonstrated the safety of this strain as well as its ability to induce humoral responses [9, 10].

*Shigella* is an intracellular microorganism that targets macrophages and gut epithelial cells; therefore, T cell mediated immunity (T-CMI) is expected to play an important role, particularly in the resolution of the disease. Despite this, only limited information is available on the role of T cells in shigellosis. In humans, production of cytokines (e.g., IFN-γ, TNF-α, IL-6, IL-4) has been demonstrated in the supernatants of PBMC of vaccinees stimulated with soluble *Shigella* antigens [11]. Additionally, production of similar cytokines has been shown at the local level in immunohistochemical studies [12–15]. However, in the case of *Shigella*, the T cell subset(s) producing these cytokines have remained largely unknown. Previous studies with CVD 1208S focused on the development of humoral immune responses. Here we focused on the ability of CVD 1208S to induce T-CMI in healthy adult volunteers following vaccination with three oral doses (28 days apart). T-CMI to *Shigella* IpaB, one of the immunogenic proteins that is part of the type 3 secretion system (T3SS) and used as a subunit vaccine candidate [16, 17], was assayed 28 days after each immunization using a novel technique developed in our laboratory. CVD 1208S was able to induce cytokine production as well as upregulation of the degranulation marker CD107a in various CD8 and CD4 memory T cell subsets. CD8 T effector memory (T_EM_) cells showed more pronounced multifunctional capacity than the other T cell subsets. The strongest T-CMI responses were detected after the first vaccine dose. The second and third vaccine doses induced responses mainly in volunteers that had not developed T-CMI to the previous vaccination(s). In sum, CVD 1208S is capable to induce T-CMI responses, which most likely complement the humoral responses elicited by this vaccine candidate and are likely to play an important role in combating *Shigella* infections.

## Methods

### Subjects and design

Healthy male and non-pregnant female volunteers aged 18–49 years were recruited from the Baltimore/Washington DC area for this randomized, placebo-controlled, double-blinded clinical trial. Volunteers were randomly allocated 3:1 to receive vaccine (n = 12) or placebo (n = 4). Each subject ingested a dose of vaccine (10^8^ CFU of CVD 1208S) or placebo on day 0, 28, and 56. Two participants did not present for the second dose. In this report we include the results of the 14 participants who completed the study (11 CVD 1208S vaccinees and 3 placebo recipients).

### Ethics statement

Written informed consent was obtained from all study participants. This study was approved by the Institutional Review Board and registered on ClinicalTrials.gov (identifier NCT01531530). The study was conducted in accordance with the principles of the International Conference of Harmonization Good Clinical Practice guidelines.

### CVD 1208S vaccine construction and preparation of a cGMP pilot lot

CVD 1208S (Δ*guaBA*, Δ*sen*, Δ*set*) was constructed from wild-type *S. flexneri* 2a strain 2457T by a series of allelic exchange reactions using suicide plasmid deletion cassette technology, as previously described [9, 18], followed by the introduction of specific in-frame deletion mutations in the chromosomal genes *guaBA* and *set* and the plasmid gene *sen* [9]. Cell banks were prepared from purified, characterized strains of CVD1208S grown in nonselective soy peptone media (Hy-Soy, Quest International, Norwich, NY). High-density growth was harvested, resuspended in soy peptone broth supplemented with 15% glycerol, and then transferred as 1.0 ml aliquots into 100 vials. The vials were sealed, rapidly frozen at − 80 °C and designated as the Master Cell Bank (MCB). A cGMP MCB was made at Paragon Bioservices, Inc., followed by transfer to the Pilot Bioproduction facility at the Walter Reed Army Institute of Research (WRAIR) for production of a cGMP pilot lot of vaccine. Each vial of lyophilized vaccine contains ~ 1.3 × 10^9^ viable cells/ml (range 0.8–2.0 × 10^9^) and is maintained at − 80 °C. Before each oral vaccine administration, total viability colony counts and proportion of Congo red colonies were measured to confirm product viability as well as stability. The lyophilized vaccine was re-suspended and diluted as needed to achieve the desired viable bacterial count per ml (optical density-O.D.). In order to estimate the actual inoculum of vaccine delivered, the replicate colony counts performed before and after vaccination were averaged. The vaccine inoculum was transported to the study site on wet ice at 2–8 °C in a vaccine transport canister and used within 4 h of preparation. Placebo consisted of buffer solution mixed with cornstarch USP to match the turbidity of the vaccine. Volunteers were not allowed to eat or drink for 90 min pre- and post-vaccination. All doses of vaccine and placebo were administered with sodium bicarbonate buffer (1.3% wt/vol).

### Clinical evaluation, bacteriological studies and humoral immunity studies

The clinical, as well as bacteriological and humoral immunity results of the volunteers who received 3 doses of 10^8^ CFU of CVD 1208S are reported in a separate manuscript currently in preparation.

### Isolation of peripheral blood mononuclear cells (PBMC)

PBMC were isolated immediately after blood draws by density gradient centrifugation and cryopreserved in liquid nitrogen following standard techniques as previously described [19, 20]. For T cell studies, PBMC from CVD 1208S and placebo recipients were isolated from blood specimens collected on day-1 (before immunization; referred as day 0 in this manuscript), and at days 28, 56 and 84.

### Construction of IpaB-LPS-nanoparticles

*Shigella flexneri 2a* lipopolysaccharide (LPS)-QDot655 micelles of nanoparticle size (approx. 30–60 nm) (LPS-nanoparticles) were generated as previously described [21–25]. The LPS from *S. flexneri* 2a used in these experiments was purified by the hot aqueous phenol extraction method of Westphal [26]. IpaB was incorporated in LPS-nanoparticles (IpaB-LPS-nanoparticles), using the LPS-nanoparticles as a vehicle to deliver IpaB to target cells. *Shigella* IpaB was purified as a recombinant protein expressed in *E. coli* [9, 27–29]. To determine incorporation of IpaB into the LPS-nanoparticle, IpaB was labeled with a Pacific Blue (PB) labeling kit (Invitrogen, Eugene, OR). To construct IpaB-LPS-nanoparticles, a master mix of LPS-nanoparticles was vortexed for 5 min and then sonicated for 15 min. The LPS concentration in the LPS-nanoparticle master mix was determined by the LAL method (Pierce, IL, USA). Subsequently, 5 µg of LPS (contained in the *S*. *flexneri* 2a-LPS-nanoparticle) were mixed with 6 µg of IpaB-PB in a total volume of 50 µl of 1% BSA and incubated at room temperature (RT) for 30 min. The IpaB-LPS-nanoparticle mixture was then vortexed for 5 min, sonicated for 5 min and incubated again for 30–60 min (RT). Integration of IpaB into the LPS-nanoparticle was evaluated by flow cytometry (Additional file 1: Figure S1).

### Stimulator cells and delivery of IpaB to stimulator cells

Autologous Epstein Barr Virus (EBV)-transformed B-lymphocytic cell lines (B-LCL) were used as stimulator cells [30, 31]. B-LCL were obtained by infection of PBMC with EBV particles [supernatant from the B95-8 cell line (ATCC CRL1612)] and cyclosporine (0.5 μg/ml; Sigma-Aldrich, Saint-Louis, MO, USA) for approximately 30 days [30, 31].

To deliver IpaB to B-LCL, one million B-LCL (100 µl of 1% BSA in PBS) were co-cultured with 50 µl of IpaB-LPS-nanoparticles (containing approximately 4–5 µg of IpaB and 5 µg of LPS) in a conical tube for 1 h (37 °C; 5% CO_2_) at room temperature. The B-LCL IpaB-LPS-nanoparticle mixture was then transferred to 24-well plates and the volume increased to 1 ml using complete media [RMPI (Gibco, NY, USA) supplemented with 10% fetal bovine serum (FBS) (Gemini Bioproducts, West Sacramento, CA), 2 mM l-glutamine (Gibco, Grand Island, NY, USA), 1× non-essential amino acids (Gibco, Grand Island, NY, USA), 10 mM HEPES (Gibco, Grand Island, NY, USA), 2.5 mM Sodium pyruvate, (Lonza, Walkersville, MD, USA), 100 U/ml penicillin, 100 µg/ml streptomycin (Sigma-Aldrich, St. Louis, MO, USA), 50 μg/ml Gentamicin (Gibco, Grand Island, NY, USA)]. B-LCL were then incubated overnight (37 °C; 5% CO_2_). Next morning, the uptake of IpaB by B-LCL (IpaB-stimulator cells) was assayed by flow cytometry. In some experiments, B-LCL were also assessed for expression of activation markers after overnight culture with IpaB-LPS-nanoparticles. For these experiments, B-LCL were stained using standard methods, as described elsewhere [24, 25], with the following monoclonal antibodies (mAbs): CD69-ECD (TP1.55.3, Beckman Coulter), CD86-PerCP-Cy5.5 (FUN-1, BD), CD40-PE-Cy7 (5C3, BD), CD80-Biotin (L307.4, BD), streptavidin (SAV)-Qdot800 (Invitrogen), and/or HLA-DR-QD605 (L243, Biolegend).

### Ex-vivo stimulation of effector cells

Cryopreserved PBMC were thawed and rested in cRPMI overnight before stimulation with IpaB-stimulator cells. B-LCL without IpaB (background stimulators) were also used to determine the background responses to EBV. Medium only and *Staphylococcus* enterotoxin B (SEB; 10 μg/ml) were used, respectively, as negative and positive controls. Stimulator cells were γ-irradiated (6000 rad) and incubated with PBMC (effector: stimulator ratio 5:1) for 2 h in the presence of anti-CD107a-FITC (clone H4A3, BD Biosciences) mAb before overnight incubation with the protein transport blockers monensin (1 μg/ml, Sigma) and brefeldin A (2 μg/ml; Sigma). Additional file 1: Figure S2 shows a summary of the assay set up.

### Immunostaining with a 13-Color mAb panel and flow cytometry analysis

Following overnight co-culture with stimulator cells, PBMC were harvested, washed in 1× PBS, stained extracellularly, permeabilized, and stained intracellularly, using a 13-color panel as previously described [24, 30, 31]. The panel used included the following mAbs: CCR7-PE (clone: G043H7, provider: BioLegend), CD4-PerCP-Cy5.5 (L200, BD Biosciences), CD19-BV421 (HIB19, Biolegend), CD3-BV650 (SP34-2, BD Biosciences), integrin α4β7-Alexa Fluor 647 (ACT1, conjugated in house), CD8-Alexa Fluor 700 (RPA-T8, BD Biosciences), CD45RA-APC-Cy7 (5H9, BD Biosciences), CD69-ECD (TP1.55.3, Beckman Coulter), IFN-γ-PE-Cy7 (B27, BD), IL-17A-BV570 (BL168, Biolegend), IL-2-BV605 (MQ1-17H12, BD Biosciences), and TNF-α-BV711 (MAb11, Biolegend). Cell viability was assessed using a Violet Live/Dead viability kit (Invitrogen, Carlsbad, CA, USA). Stained cells were fixed with 1% PFA in PBS. Samples were acquired using a customized LSRII flow cytometer (BD Biosciences) and analyzed using Flowjo v10 (FlowJo, LLC, San Francisco, CA). Responses against IpaB (*S. flexneri* 2a) were expressed as net percentage of positive cells (i.e., total percentage of positive cells in the presence of IpaB-stimulating cells (targets) minus percentage of positive cells in co-cultures with background stimulators (B-LCL without IpaB).

### Evaluation of necrosis and apoptosis in B-LCL and immunostaining for activation markers

Necrosis and apoptosis of B-LCL following exposure to IpaB alone or IpaB-LPS-nanoparticles was assessed using Annexin V labeled with APC (BD Biosciences) and yellow fixable dye (YeVID) (Invitrogen). For these experiments one million B-LCL (100 µl) were cultured either with LPS-IpaB-nanoparticles (containing approximately 4–5 µg of IpaB and 5 µg of LPS) or IpaB-PB alone (approximately 5 µg) for 1–2 h. Subsequently, cells were stained in 96-well plates as previously described [24, 25]. Briefly, cells were washed with 2× with PBS, stained with YeVID (15 min) and subsequently washed 2× with a 4% BSA-PBS solution. Then, the cells were washed twice in 1× Annexin V binding buffer (BD Biosciences) and incubated with Annexin V-APC prepared in 1× Annexin V binding buffer. Cells were stained for 15 min at room temperature and after washing the cells once again, the samples were immediately acquired using a customized LSRII flow cytometer (BD Biosciences).

To assay for upregulation of activation markers, B-LCL were exposed to LPS-IpaB-nanoparticles for 16–18 h. Cells were then stained in 96-well plates using the following mAbs: CD69-ECD (TP1.55.3, Beckman Coulter), CD80-Biotin (L307.4, BD Biosciences), CD40-Pe-Cy7 (5C3, BD Biosciences) CD86-PerCp-Cy5.5 (FUN-1, BD Biosciences), HLA-DR-QDot605 (Tü36, Invitrogen) and Streptavidin-Pacific Orange (Invitrogen). Cell viability was assessed using a Violet Live/Dead viability kit (Invitrogen, Carlsbad, CA, USA). Stained cells were fixed with 1% PFA in PBS and analyzed as described above.

### Statistical methods

The percentage of (net percentage) multifunctional (MF) and single function (S+) cells as well as the various MF populations (2+ to 5+) in the different CD4 and CD8 memory subsets were evaluated using the Mann–Whitney comparison test. Changes in expression of integrin α4β7 expression following vaccination were evaluated via one way ANOVA followed by Tukey’s multiple comparison test. Microsoft Excel and GraphPad Prism were used for statistical analysis. All hypotheses were evaluated using two-sided tests. Two-sided P values < 0.05, without adjustment for multiple comparisons, were considered statistically significant.

## Results

### IpaB-induced necrosis in B-LCL is reduced when IpaB is delivered by LPS-nanoparticles

**Fig. 1:**
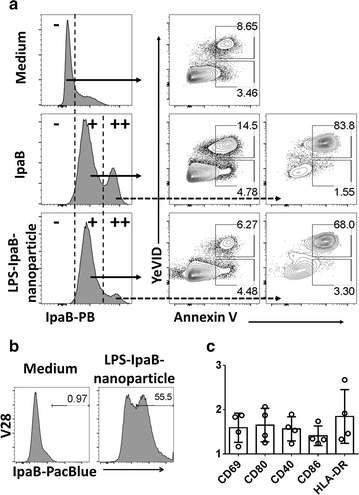
Delivery of IpaB to B-LCL cells by LPS-nanoparticles. **a** Induction of necrosis/apoptosis in B-LCL by IpaB. Top panels: B-LCL exposed to media for 2 h. Center panels: in B-LCL exposed for 2 h to IpaB (Pacific Blue) two populations were identified (IpaB+ and IpaB++), IpaB+ B-LCL showed an increased in the percentage of cells undergoing necrosis (YeVID+, Anexin V+) (~ twofold compared to media), but no changes in the percentage of cells undergoing apoptosis (YeVID−, Anexin V+). IpaB++ cells showed an important increase in necrosis (~ tenfold compared to media), but not in apoptosis. Bottom panels: in B-LCL exposed for 2 h to to IpaB incorporated into the LPS-nanoparticle (LPS-IpaB-nanoparticles) the same two populations were identified (IpaB+ and IpaB++). However, no induction of necrosis was seen in the IpaB+ population. Also, the IpaB++ population showed a lower degree of necrosis than when exposed to IpaB alone. The data shown is from one representative volunteer of 4 evaluated. **b** After 18 h of incubation with the LPS-IpaB-nanoparticles, IpaB can be identified in 50–60% of B-LCL and that these cells (**c**) display upregulation of activation markers. Data in **b** is from one representative volunteer (#28) and data in **c** is a composite from four different vaccinees assayed in different days (#39, 24, 30 and 40)

To evaluate T cell mediated immune responses (T-CMI), we used autologous B-LCL as antigen presenting cells (stimulator/target cells). B-LCL were generated by transforming B cells with EBV. In initial experiments, cell death was noted in B-LCL from various volunteers when exposed to IpaB (4–5 µg; 1–2 h). We investigated whether the cell death induced by IpaB in B-LCL was due to necrosis or apoptosis using a dual staining method with a viability dye (YeVID) (to evaluate integrity of the cell membrane) and Annexin V (to evaluate translocation of phosphatidylserine) (Fig. 1a). We observed that IpaB induced necrosis (YeVID+ Annexin V+) but not apoptosis (YeVID− Annexin V+) (Fig. 1a middle panels). Interestingly, cells that incorporated higher levels of IpaB (IpaB++) had significantly more necrosis that those that incorporated less IpaB (IpaB+). Various strategies to reduce IpaB-induced necrosis were evaluated, including the delivery of IpaB to B-LCL using LPS-nanoparticles (Fig. 1a bottom panels and Additional file 1: Figure S1). Interestingly, the delivery of IpaB by the LPS-nanoparticle resulted in no increase in necrosis (compared to B-LCL exposed to media); of note, in those cells that incorporated high levels of IpaB (IpaB++) the percentage of necrotic cells was somewhat reduced (Fig. 1a, bottom panels). Moreover, after overnight stimulation with LPS-IpaB-nanoparticles, upregulation of activation markers in cells that were IpaB+ (Fig. 1b, c) was noted. Thus, we decided to use this approach in subsequent experiments to deliver IpaB to B-LCL cells and use them as stimulator/target cells to evaluate T cell mediated immunity (T-CMI) to *Shigella* IpaB.

### T-CMI induced in T effector memory (T_EM_) cells by CVD 1208S

**Fig. 2:**
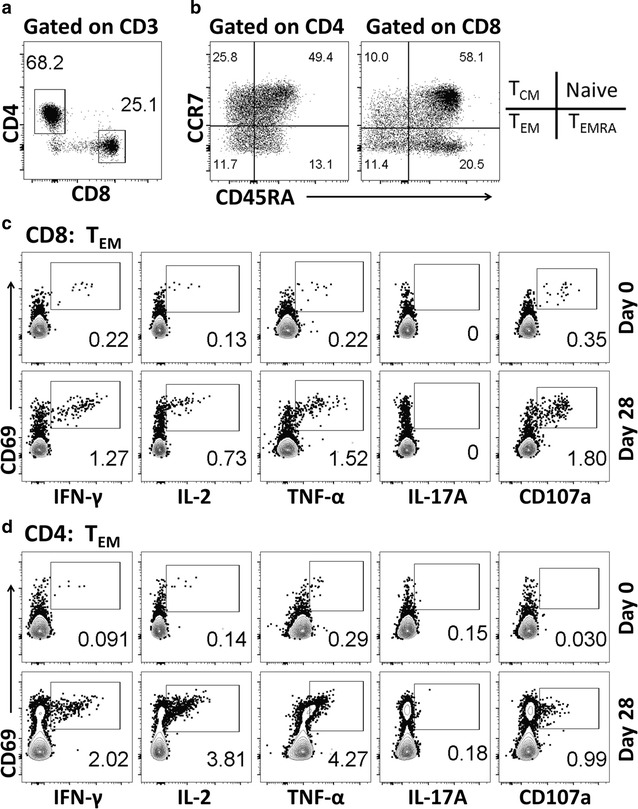
Gating strategy used for the identification of cytokine producing T cells. **a** The gating strategy used to identify CD4 and CD8 T cells. **b** displays the memory subsets defined by CCR7 and CD45RA in CD4 and CD8 T cells. **c**, **d** Examples of cytokine upregulation (Pre and post vaccination, days 0 and 28 respectively) in CD8 and CD4 T effector memory (T_EM_) cells of a representative vaccinated volunteer

PBMC from CVD 1208S and placebo volunteers were evaluated for their ability to produce cytokines (IFN-γ, IL-2, IL-17A, TNF-α) or upregulate the degranulation marker CD107a at multiple time points before (day 0) and 28 days after each vaccination. T-CMI was evaluated using high-color flow cytometry (13-color panel) that allowed simultaneous evaluation of the markers described above in various CD4 and CD8 T cell subsets (CCR7/CD45RA classification). Additionally, this panel allowed us to explore expression of the gut homing marker integrin α4β7 in the T cell subsets. Figure 2 shows an example of the gating strategy used as well as the different T cell subsets defined by CCR7/CD45RA [Naïve: CCR7+CD45RA+; T central memory (T_CM_): CCR7+CD45RA−; T effector memory (T_EM_): CCR7−CD45RA−; and T effector memory CD45RA positive (T_EMRA_): CCR7−CD45RA+].

**Fig. 3:**
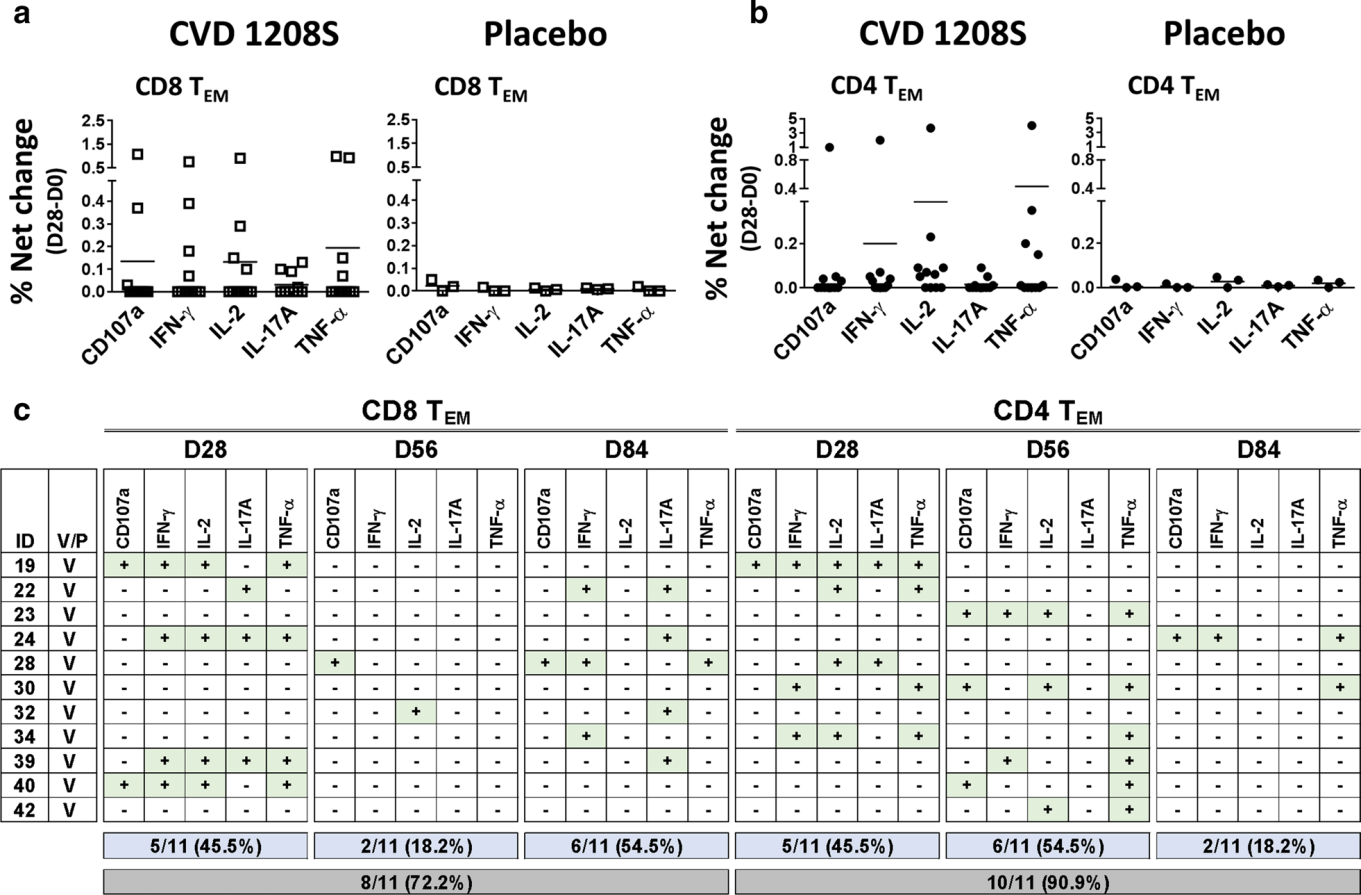
CD107a upregulation or cytokine production by T_EM_ cells after CVD 1208S immunizations. **a** Displays CD107a upregulation [% net change (day 28-day0)] (degranulation marker) or cytokine production by CD8 T_EM_ cells in CVD 1208S (left panel) and placebo (right panel) recipients 28 days after the first immunization. In **b** similar data is presented for CD4 T_EM_ cells. **c** A summary of CD107a expression or cytokine production 28 days after each immunization (days 28, 56 and 84) in each vaccinated individual. The data is shown for CD8 and CD4 T_EM_ cells. Volunteers who upregulated CD107a or exhibited increases in particular cytokines are indicated with a + sign in a green box (data derived from % net changes relative to day 0). Indicated in the blue boxes are the number (and percentage) of vaccinated volunteers that exhibited increases in at least one cytokine or upregulated CD107a after each immunization. The gray boxes show the number and corresponding percentage (%) of vaccinated volunteers that exhibited increases in at least one cytokine or upregulated CD107a after receiving the three vaccine doses

The main subsets producing cytokines to bacterial and viral antigens are CD8 and CD4 T_EM_ cells. Therefore, these were the first subsets evaluated (Fig. 3). Before vaccination, no differences in cytokine production or CD107a expression between CVD 1208S and placebo recipients in CD8 T_EM_ and CD4 T_EM_ cells were noted (Additional file 1: Figure S3). Twenty-eight days after the initial vaccination, upregulation of CD107a and increased production of IFN-γ, IL-2, IL-17A, and TNF-α were detected in CD8 T_EM_ and CD4 T_EM_ cells from various CVD 1208S recipients (Fig. 3a, b). Importantly, increased production of these cytokines or CD107a upregulation were not detected in placebo recipients. To define positive increases in cytokine production or CD107a expression, the net percentages [percentages of a given cytokine on day “X” post-vaccination minus levels at day 0 (pre-vaccination)] in the placebo volunteers at all time points (3 time points) were collected and averaged, and a fourfold increase above the arithmetic mean was considered positive. For example, using this approach we determined that on day 28 of the study, in CD8 T_EM_ cells 2 of 11 (18.2%) vaccinated volunteers upregulated CD107a, 4 (36.4%) expressed IFN-γ, IL-2 and TNF-α and 3 (27.3%) expressed IL-17A. Additional file 1: Table S1 summarizes the number and percentage of volunteers that produced increased cytokines or upregulated CD107a at different time points in the various T cell subsets studied.

**Fig. 4:**
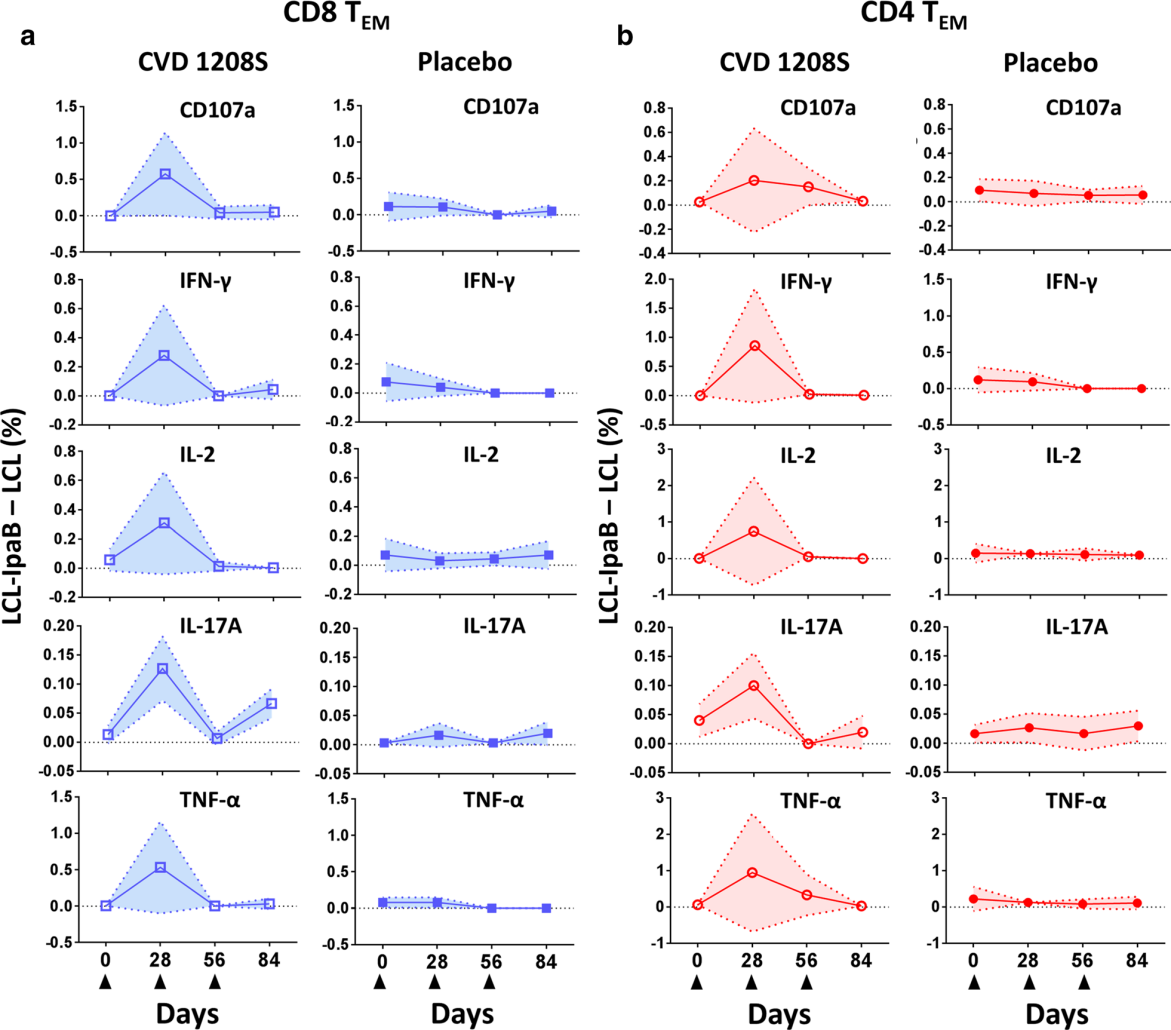
Kinetics of cytokine production or CD107a expression in T_EM_ cells. **a** Kinetics of CD107a expression (degranulation marker) or cytokine production by CVD1208S (left panels) and placebo (right panels) recipients in CD8 T_EM_ cells. Filled triangles at the bottom indicate vaccination days. In the vaccinated group only the data of the individuals that showed increase in CD107a or cytokine expression relative to pre-vaccination (Fig. 3c) were used to generate these graphs. In the placebo group all the available data was used. The data is presented as the difference between the percentage of B-LCL cells loaded with IpaB and unstimulated B-LCL cells [LCL-IpaB − LCL (%)]. **b** Similar data from CD4 T_EM_ cells. Arithmetic mean ± SD are displayed in the plots

CD8 T_EM_ cell data showed that the same volunteers were not upregulating similar cytokines (Fig. 3c). Moreover, the stronger and more diverse immune responses were induced by the first vaccination (Figs. 3c, 4a). Subsequent, immunizations induced responses but mainly in volunteers who had not responded to the previous vaccination and the diversity (more than one cytokine produced) was lower. For example, on CD8 T_EM_ cells, 5 volunteers (45.5%) produced cytokines after the first immunization. Of these, 4 volunteers (#19, #24, #39 and #40) produced more than 1 cytokine. However, after the second immunization (analyzed on day 56), only 2 volunteers (#28 and #32) produced cytokines and these CD8 T_EM_ cells were single-cytokine producers. After the third immunization (analyzed on day 84), 6 volunteers showed responses, of these only 2 (#22 and 28) showed production of multiple cytokines. One of the volunteers on day 84, had not responded previously to CVD 1208S (#34), and the responses in this volunteer was of single cytokine production.

Similar analysis was performed on CD4 T_EM_ cells (Fig. 3b, c, 4b and Additional file 1: Table S1). The first immunization was the one that induced the strongest responses (Fig. 4b); however, diversity appeared to be similar after the first and second vaccinations since 5 volunteers showed more than 1 cytokine being produced after each one of these doses. Importantly, of the 6 volunteers that showed responses after the second vaccination, 4 were non-responders to the first dose. After the third vaccination, only 2 volunteers showed responses, of these 1 (#24) had not showed CD4 TEM responses to the previous vaccine doses.

Once the three doses of the vaccine course were completed, in CD8 T_EM_ and CD4 T_EM_ cells 8 (72.2%) and 10 (90.9%) volunteers, respectively, showed at least one cytokine being produced at some point after immunization. Despite that the first vaccine dose induced the strongest T-CMI, subsequent vaccinations were important for previous non-responders.

### Multifunctional (MF) T_EM_ cells induced by CVD 1208S

**Fig. 5:**
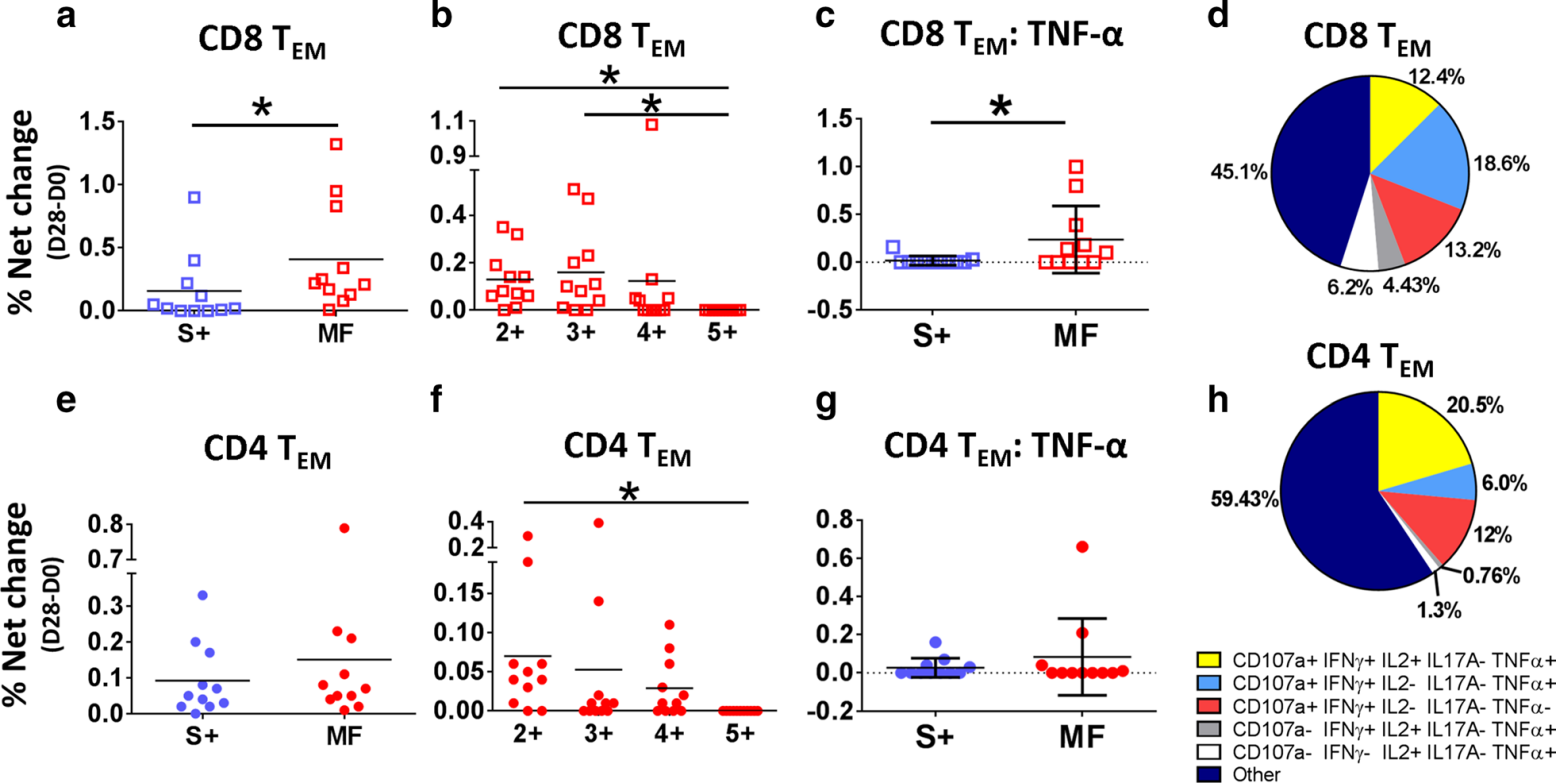
Multifunctional responses induced by CVD 1208S in T_EM_ cells 28 days after the first immunization. **a**, **e** The comparison of cell that showed a single function (S+) (e.g., cells producing one cytokine or upregulating CD107a alone) versus multifunctional (MF) cells (e.g., cells exhibiting more than one function) in CD8 and CD4 T_EM_ cells, respectively. **b**, **f** The comparison of cells that showed 2–5 simultaneous functions (2+, 3+, 4+ and 5+). For each cytokine, S+ and MF cells were compared. **c** CD8 T_EM_ MF cells that produced TNF-α (in any combination) were more abundant than those producing TNF-α alone. These results were not observed in CD4 T_EM_ cells (**g**). The arithmetic mean ± SD is indicated in all the groups. **d**, **h** The relative frequency (expressed as percentage) of the 5 most prevalent MF populations in CD8 T_EM_ and CD4 T_EM_ cells, respectively. The remaining MF populations were pooled in a single group indicated as other. *P < 0.05 (Mann–Whitney)

After exploring whether individuals produced one or more cytokines/CD107a (henceforth referred as functions) (Fig. 3), we examined whether individuals in whom more than one function was detected (e.g., #19; Fig. 3c) elaborated simultaneously more than one cytokine/CD107a (multifunctional -MF-). MF cells were analyzed using Boolean gates (Flowjo). Importantly, since the first vaccine dose induced the most diverse responses in CD8 T_EM_ cells, analyses involving MF cells were performed only at this time point. Initially, we explored whether cells producing a single cytokine/CD107a (single positive; S+) were as abundant as MF cells. S+ were considered cells producing any cytokine/CD107a, while MF involved cells with more than one function (any combination of functions). Among CD8 T_EM_ cells, MF cells were significantly more abundant than S+ cells (Fig. 5a). Later, among MF cells we explored whether any population of cells that showed 2, 3, 4, or 5 functions (referred as 2+, 3+, 4+ and 5+, respectively), in any combination, was more abundant than the other. The results showed that 2+ and 3+ cells were the most abundant and cells with 5 functions were virtually absent (Fig. 5b). Considering that MF cells were more abundant than S+ cells, we then explored whether S+ cells expressing a specific cytokine were more abundant than MF+ expressing the same cytokine (in any combination). The results showed that MF cells expressing TNF-α were more abundant than cells producing TNF-α alone (S +) (Fig. 5c). Finally, we looked at the 5 most frequent MF combinations (data presented as percentage of a whole) (Fig. 5d). The MF combinations that were not part of the top 5 populations were combined in a single group denominated “Other”. Among the 5 most frequent MF combinations, 4 of them included TNF-α; however, no population was significantly predominant.

Similar analyses were performed in CD4 T_EM_ cells. Interestingly, despite the finding in the previous analyses that CD4 T_EM_ cells produced various cytokines (e.g., #19, #22, etc.; Fig. 3c), S+ and MF cells were equally abundant (Fig. 5e). Among MF cells, 2+ were the most frequent (Fig. 5e). Unlike CD8 T_EM_ cells, MF cells that expressed TNF-α were not predominant. In fact, no cytokine (in any combination) was predominant in MF cells. Since no cytokine predominance in MF populations was noted, the same MF populations analyzed in CD8 T_EM_ cells were analyzed in CD4 T_EM_ cells. As expected, the cumulative percentage of the top 5 MF population analyzed was ~ 40%, while the “Other” population was ~ 60% providing more evidence for the lack of dominance of a specific MF population (cytokine combination).

### T-CMI in T_EMRA_ and T_CM_ populations

**Fig. 6:**
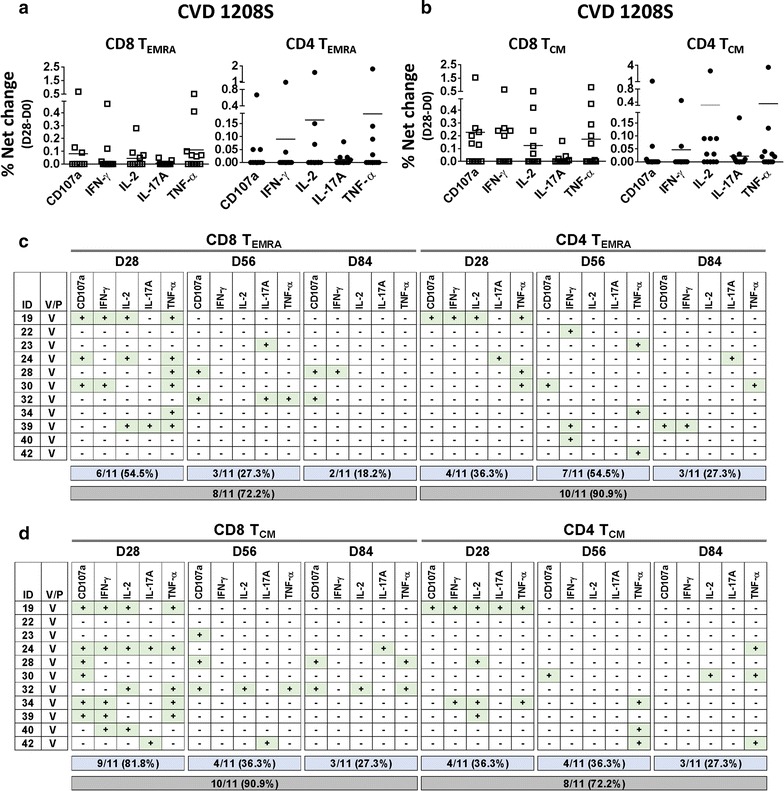
Upregulation of CD107a and cytokine production by T_EMRA_ and T_CM_ cells after CVD 1208S immunizations. **a** CD107a upregulation [% net change (day 28 minus day0)] or cytokine production by CD8_EMRA_ (left panel) and CD4T_EMRA_ (right panel) cells in CVD 1208S recipients 28 days after the first immunization. **b** Similar data are presented for CD8 and CD4 T_CM_ cells. **c**, **d** A summary of CD107a upregulation or cytokine production 28 days after each immunization (days 28, 56 and 84) in each vaccinated individual. The data shown corresponds to CD8 and CD4 T_EMRA_, as well as CD8 and CD4 T_CM_ cells. Volunteers who upregulated CD107a or produced cytokines are indicated with a + sign in a green box (data are derived from % net changes relative to day 0). In the blue boxes the number (and percentage) of vaccinated volunteers that produced at least one cytokine or upregulated CD107a after each immunization are indicated. The gray boxes indicate the number (and percentage) of vaccinated volunteers that exhibited increases in at least one cytokine or upregulated CD107a after receiving the three vaccine doses

We subsequently analyzed T-CMI in the other major CD4 and CD8 memory T cell subsets. We identified responses in both CD8 T_EMRA_ and CD4 T_EMRA_ cells of CVD 1208S recipients (Fig. 6a and Additional file 1: Table S1). These responses were absent in placebo volunteers (Additional file 1: Figure S3B). Similar to T_EM_ cells, the first immunization induced stronger and more diverse responses in CD8 T_EMRA_ and CD4 T_EMRA_ cells than subsequent vaccine doses (Fig. 6c). The second and third doses were important in inducing responses in previous non-responders. Following the first, second and third immunization in 6 (54.5%), 3 (27.3%) and 2 (18.2%) of 11 CVD 1208S recipients, respectively, exhibited increases in at least one cytokine/CD107a in CD8 T_EMRA_ cells. The cumulative data, showed that after completing the 3 vaccine doses, 8 of 11 volunteers (72.2%) produced at least one cytokine/CD107a (Fig. 6c). On CD4 T_EMRA_ cells, after each vaccine dose, 4 (36.3%), 7 (54.5%) and 3 (27.3%) of the 11 vaccinated volunteers showed increases in at least one cytokine/CD107a, with a cumulative data of 10 of 11 volunteers (90.9%) exhibiting responses.

Cytokine production or CD107a upregulation to *Shigella* IpaB were also identified in CD8 T_CM_ and CD4 T_CM_ cells of vaccinees (Fig. 6b and Additional file 1: Table S1), but absent in placebo volunteers (Additional file 1: Figure S3B). Interestingly, the responses in CD8 T_CM_ cells were particularly robust and diverse after the first vaccine dose since 9 of 11 volunteers (81.8%) of the individuals showed increases in at least one cytokine/CD107a. Similar to the other populations, second and third vaccinations resulted in less robust (36.3 and 27.3% of vaccinees, respectively) and diverse responses (Fig. 6d). After receiving the three CVD 1208S doses CD8 T_CM_ cells of 10 of 11 (90.9%) of individuals exhibited increases in cytokines/CD107a. On CD4 T_CM_ cells, the responses were less robust than on CD8 T_CM_ cells, but the same pattern of stronger and more diverse responses after the first immunization than subsequent ones was observed. After three CVD 1208S doses, CD4 T_CM_ cells of 8 of 11 (72.2%) volunteers exhibited increases in at least one cytokine/CD107a (Fig. 6d).

### Multifunctional responses in T_EMRA_ and T_CM_ populations

**Fig. 7:**
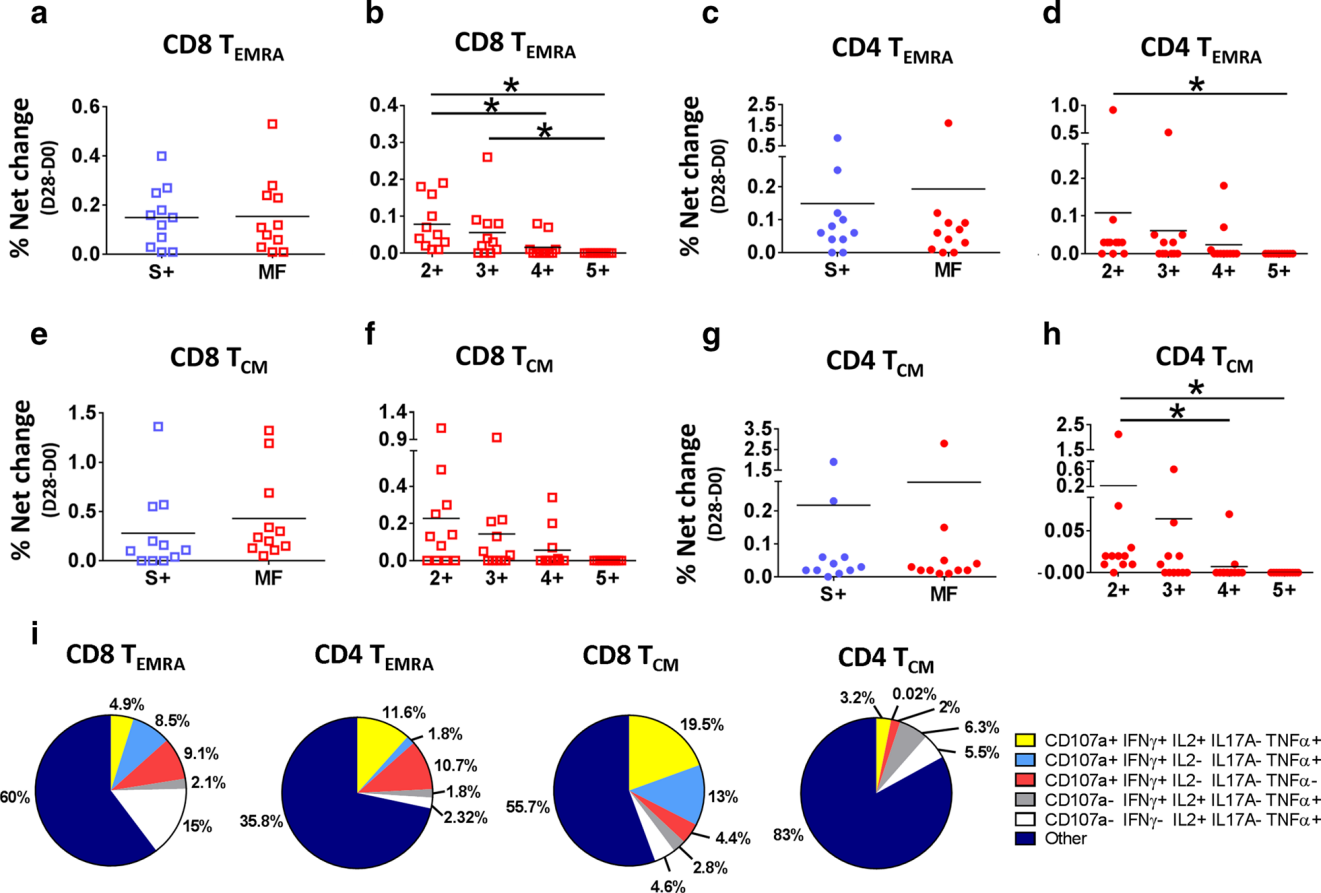
T_EMRA_ and T_CM_ cells responses to CVD 1208S, 28 days after first immunization. **a**, **c**, **e**, **g** The comparison of single function (S+) versus multifunctional (MF) CD8 T_EMRA_, CD4 T_EMRA_, CD8 T_CM_, and CD4 T_CM_ cells respectively, in response to CVD1208S immunization. **b**, **d**, **f**, **h** display the comparison of cells that showed more than one simultaneous function (e.g., increased cytokine production or CD107a upregulation) in CD8 T_EMRA_, CD4 T_EMRA_, CD8 T_CM_, and CD4 T_CM_ cells, respectively. Means are indicated in each graph. *P < 0.05 (Mann–Whitney). **i** The relative frequency (expressed as percentage) of the 5 most prevalent MF populations in CD8 T_EMRA_, CD4 T_EMRA_, CD8 T_CM_, and CD4 T_CM_ cells. The remaining MF populations were pooled in a single group indicated as other

Induction of MF responses in T_EMRA_ and T_CM_ cells were explored in both CD8 and CD4 populations (Fig. 7). Also, considering that the first immunization induced the most diverse and robust T-CMI responses, multifunctionality in T_EMRA_ and T_CM_ cells was assessed only in this time point (day 28 of the study). In contrast to the observations with CD8 T_EM_ cells, no differences in the frequency between S+ and MF (any function combination) cells were detected in CD8 T_EMRA_, CD4 T_EMRA_, CD8 T_CM_ and CD4 T_CM_ cells (Fig. 7a, c, e, g, respectively). Among MF cells, 2+ and 3+ (any function combination) were the most abundant in CD8 T_EMRA_, CD4 T_EMRA_, CD8 T_CM_ and CD4 T_CM_ cells (Fig. 7b, d, f, h, respectively). Finally, since no cytokine predominated among MF cells, we evaluated the same 5 populations assessed in CD8 T_EM_ cells (Fig. 7i). The results showed that similar to CD4 T_EM_ cells the “Other” MF population was the most abundant (> 50%) in every case explored, confirming that no MF population predominated.

### Upregulation of integrin α4β7 in CD8 T_EM_ cells in CVD 1208S vaccinees

**Fig. 8:**
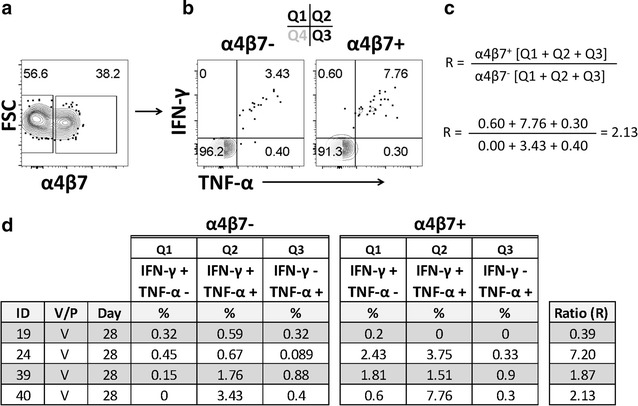
Increased cytokine production by CD8 T_EM_ cells expressing integrin α4β7 among CVD 1208S vaccinees. **a** Identification of CD8 T_EM_ cells expressing integrin α4β7. **b** Gating example to evaluate the expression of IFN-γ and TNF-α in integrin α4β7− and integrin α4β7+ CD8 T_EM_ cells among individuals who exhibited increased production of these cytokines after the first immunization (day 28). The data shown in **b** are from volunteer #24. **c** Formula used to calculate the ratio (R) of cytokine production (IFN-γ and TNF-α) among integrin α4β7− and integrin α4β7+ CD8 T_EM_ cells. **c** Also displays an example of the calculations using data from volunteer #24, i.e., each of the 3 quadrants (Q1, Q2 and Q3) containing single or double positive cells). **d** A summary of the data in integrin α4β7− and integrin α4β7+ in CD8 T_EM_ cells from the vaccinated individuals that increased TNF-α and IFN-γ 28 days after the first vaccination

*Shigella* is a gut restricted pathogen; therefore, immune responses at the local level (gut mucosa and submucosa) are expected to be predominant. Importantly, CVD 1208S is an oral vaccine and therefore, evaluation of whether CVD 1208S induces upregulation of the gut homing molecule integrin α4β7 in cells in the periphery (PBMC), particularly those producing cytokines in response to IpaB was determined. We focused our analysis in CD8 T_EM_ cells because these showed diverse responses and were the only ones that showed MF capacity. Additionally, the analysis involved only day 28 since this is the time point with the strongest responses. Among CD8 T_EM_ cells, we identified cells producing integrin α4β7 and then we evaluated the expression of TNF-α and IFN-γ (Fig. 8). TNF-α was selected because increased production of this cytokine was associated with MF capacity. Additionally, IFN-γ was selected because a body of literature has consistently reported increased production of this cytokine following vaccination/challenge with various *Shigella* strains [9, 11, 32, 33]. These analyses were therefore performed only on those individuals who showed significant increase in these cytokines at day 28 (Fig. 3c; volunteers 19, 24, 39 and 40). To determine whether cells expressing integrin α4β7 produced higher levels of the cytokines, we generated a ratio (R) between integrin α4β7+ and integrin α4β7− cells producing these cytokines (Fig. 8c). The results summarized in Fig. 8d demonstrate that in three of the four volunteers (volunteers 24, 39 and 40) integrin α4β7+ cells produced higher levels of cytokines than integrin α4β7− cells (R > 1).

## Discussion

Even though it is widely accepted that serotype-specific humoral responses play an important role in protection from *shigellosis* [3, 29, 34], the intracellular nature of this microorganism suggest that T-CMI might also play a significant role, particularly in ameliorating the severity and duration of symptoms. Evaluation of these immune responses in humans has been limited [12, 35]; however, some studies have suggested a role for T-CMI in shigellosis. For example, mononuclear cells from some volunteers challenged with a Shiga toxin-deleted *S. dysenteriae* 1 strain SC595 exhibited increased production of IFN-γ and IL-10 after challenge [11]. IFN-γ was also detected in the culture supernatants of PBMC from some human volunteers vaccinated with the *S. sonnei* (WRSS1) and *S. flexneri* 2a (CVD 1207) candidate strains [32, 33]. Moreover, IFN-γ production by PBMC following ex vivo stimulation with IpaB (ELISpot) was reported in recipients of the *S. flexneri* 2a vaccine candidate CVD 1208S [9]. The last report is of particular importance, since IFN-γ production was detected in 25 and 57% of volunteers receiving a single dose of either 10^8^ and 10^9^ CFU, respectively, of CVD 1208S. In the current study, after the first dose of CVD 1208S (10^8^ CFU) IFN-γ production was detected in CD8 T_EM_, T_EMRA_ and T_CM_ in 34.6, 18.2 and 45.5%, respectively, of the vaccinated volunteers. These percentages fall within the expected responses for this vaccine at this dose. Initial studies showed cytokine production by PBMC in humans after *Shigella* vaccination, but the cell populations producing these cytokines remained unknown. Therefore, in the current study we further characterized the T cell subsets responsible for cytokine production after *Shigella* vaccination as well as whether these cells exhibited multifunctional properties.

Our initial attempts to evaluate T-CMI to IpaB resulted in cell death of autologous B-LCL from various volunteers. *Shigella*-induced cell death due to necrosis and apoptosis has been reported by various groups in several cell types (e.g., macrophages, neutrophils, B cells) [36–38]. IpaB-induced apoptosis in macrophages has been described and the mechanism identified [39]. Induction of necrosis by *Shigella* in human neutrophils has also been reported. This process was dependent on IpaB and IpaC and involved actin polymerization [36]. However, the mechanism by which IpaB induces necrosis in B-LCL remains unknown. A possible molecule to consider is CD44, the receptor for hyaluronic acid and a widely distributed cell surface glycoprotein that exist in a variety of isoforms. CD44 has been implicated in a number of cellular adhesion processes, as well as cell growth, differentiation, modulation of apoptosis and necrosis [40]. It was recently reported that the binding of the inflammatory mediator ultra-low-molecular-weight hyaluronan (ULMW-HA) to CD44 in B-precursors leukemia cells resulted in necrosis [37]. The degree of necrosis induction depended on the level of CD44 expression; cells that expressed high levels of CD44 had higher rates of necrosis. Importantly, CD44 has been reported as a receptor for *Shigella* IpaB in epithelial cells [41, 42]. Moreover, CD44 is expressed on B cells [43–45]. It is reasonable to speculate that if CD44 is involved in induction of necrosis in B-LCL, incorporation of IpaB into LPS-nanoparticles is likely to mask the CD44-biding site of IpaB. Another aspect to consider is that bacterial LPS is composed of large repetitive polysaccharide chains which facilitate simultaneous binding of multiple B cell receptors (BCRs). For this reason, LPS is considered a T-cell-independent type 1 (TI-1) antigen [46], capable of eliciting strong polyclonal B cell activation due to the repetitive cross-linking of multiple BCRs. Incorporation of IpaB into LPS-nanoparticles might facilitate uptake of IpaB by B-LCL, using the TI-1 route while bypassing CD44-binding and therefore limiting necrosis. The use of fluorescently labeled IpaB allowed verification of its uptake by B-LCL. In sum, the approach to deliver IpaB in the context of LPS-nanoparticles to B-LCL reduced necrosis and facilitated their activation (Fig. 1) enabling the use of these cells as stimulators/targets in T-CMI assays.

Once this method to produce stimulator/target cells was developed and optimized, we evaluated the induction of CD4 and CD8 T-CMI by determining cytokine induction (IFN-γ, IL-2, IL-17A, TNF-α) or CD107a upregulation in various T memory subsets (Fig. 2). Overall, CVD 1208S induced T-CMI responses in all the CD8 and CD4 T cell memory subsets assessed. The first vaccine dose induced the strongest and most diverse responses in both CD8 and CD4 T cell subsets (Figs. 3, 4, 6). The second and third doses also induced T-CMI responses; however, these were mainly in volunteers who had not shown T-CMI responses to the previous doses. No booster effect was noted. Overall, after receiving the three vaccine doses, production of at least 1 cytokine was reported in 8–10 of 11 (72.2–90.9%) vaccinated volunteers in the CD8 and CD4 T cell subsets (Figs. 3c, 6c, d). The reason for the lack of booster responses after the second and third vaccine doses is unclear. However, it can be speculated that for this particular vaccine, the window of time between the doses was not optimal. In non-human primate studies involving malaria vaccine candidates, it was shown that extending the time between vaccine doses from 4 to 8 weeks boosted the malaria-specific CD8 T cell responses that were associated with protection from wild-type challenge [47]. Similarly, in hepatitis B vaccine studies, the immunity to this vaccine was enhanced in several volunteers receiving a delayed third vaccine dose [48, 49]. Therefore, spacing of the vaccine doses might provide an additional tool to enhance the response to CVD 1208S.

CD8 T_EM_ and CD8 T_CM_ cell showed the strongest and most diverse responses to CVD 1208S after the first dose. However, only CD8 T_EM_ cells showed a higher frequency of MF than S+ cells. Interestingly, in CD8 T_EM_ cells TNF-α was detected only in cells that produced another cytokine or expressed CD107a; therefore, TNF-α+ cells were mainly MF (Fig. 5c). Moreover, 4 of the 5 predominant MF populations expressed TNF-α, providing more evidence of the importance of this cytokine (Fig. 5d). It is also important to note that previous studies identified IFN-γ as one of the main cytokines induced by live-attenuated *Shigella* vaccines [11, 32, 33]. In the current study, we confirmed IFN-γ induction by CVD 1208S and showed that this cytokine was increased in 4 of the 5 predominant MF populations. TNF-α and IFN-γ are inflammatory cytokines and the latter plays an important role in the resistance to infection by *Shigella* in the mouse model [50]. One of the mechanisms of resistance involves inhibition of *Shigella* replication via the cytoplasmic RNA sensor retinoic acid-inducible gene I (RIG-I) [51]. The mechanism by which TNF-α limits *Shigella* infection is still unclear; however, this cytokine is known to play a critical role controlling other intestinal infectious bacteria in the mouse model [52]. MF cells appear to be of particular importance for control of disease progression in HIV and other diseases [53–56]. Whether the same principle applies to *Shigella* remains to be explored, but if we assume that this is the case, the induction of CD8 T_EM_ cells expressing TNF-α and/or IFN-γ along with other cytokines suggest that the MF immunity induced by CVD 1208S could aid in controlling infection of *Shigella*. Pioneering experiments in rectal biopsies from convalescent patients showed cells producing cytokines [14, 15]; demonstrating the induction of T-CMI at the intestinal level. Since CVD 1208S is a live-attenuated oral vaccine strain able to invade epithelial cells, we expect this vaccine to induce robust local T-CMI responses mimicking those induced by wild-type *Shigella*.

Despite the fact that CD8 T_CM_ cells also showed strong and diverse responses, MF cells in this compartment were less frequent and no cytokine showed predominance as TNF-α did in CD8 T_EM_ cells. CD4 T-CMI responses in all the T memory subsets analyzed (T_EM_, T_EMRA_ and T_CM_) followed the same pattern than CD8 T_EM_ cells responses, but showed neither MF dominance, nor the same degree of importance for TNF-α production. The diverse T-CMI detected in the different CD8 and CD4 memory subsets reflect the complexity of the responses to *Shigella* and probably also demonstrate the different kinetics of these cell populations. The current study provides evidence of the MF nature of T-CMI systemic responses. Moreover, since CD4 T-CMI responses, which are critical for the development of humoral responses are present, it is likely that CVD 1208S induces long term memory.

Of note, we also evaluated expression of CD107a on the surface of the cells. CD107a and b are normally present inside the cell in the membrane of cytotoxic granules. Mobilization of CD107a and b to the surface of the cells is dependent on degranulation and therefore an indication of cytotoxic activity (CTL) [57, 58]. CTL activity has been reported in both CD8 and CD4 T cells [59]. In the current study we showed induction of CTL activity (CD107a expression on the surface of the cell) by CVD 1208S in all the subsets of CD8 and CD4 cells (T_EM_, T_EMRA_ and T_CM_ subsets). As expected CD8 cells showed stronger CTL activity than CD4 T cells and among the subsets, CD8 T_CM_ cells showed a higher number of volunteers with CTL activity [6 of 11; 55% (Fig. 6d)] after the first vaccine dose, suggesting that in the case of *Shigella*, this subset could play an important role limiting disease progression. Additionally, in CD8 T_EM_ cells, CTL was identified in 2 of the 5 predominant MF groups; suggesting that CTL activity in combination with other cytokines (e.g., IFN-γ and TNF-α) could have an important role limiting shigellosis. This is, to our knowledge, the first report showing induction of CTL activity by a live-attenuated oral vaccine to *Shigella* and suggest that CTL could be important in eliminating *Shigella*-infected cells.

During the development of immune responses, primed cells migrate to secondary lymphoid organs. Some of these cells are identified in the peripheral blood. Therefore, PBMC provide a window to explore some of the responses that are being developed at the local (gut) level. Nevertheless, the responses in the periphery are expected to be of smaller intensity, and perhaps different in their characteristics, than at the local level. Of particular importance as a first step in studying this phenomena is the measurement of the expression of gut homing molecules, such as integrin α4β7, which suggests that the immune cells measured systemically are in the process of migrating to the gut. Importantly, we demonstrated that CD8 T_EM_ cells expressing integrin α4β7 showed enhanced cytokine production (TNF-α and IFN-γ) than those cells that were integrin α4β7− (Fig. 8). This was detected in three of four individuals (75%) that had increased expression these cytokines 28 days after the first vaccine dose. This clearly suggested that IpaB specific CD8 T_EM_ cells continued to migrate to the gut 4 weeks after vaccination. In the individual that showed the higher cytokine production of cytokines in integrin α4β7− cells, it is likely that most of the IpaB specific cells had already migrated to the gut. This would be in line with previous studies in individuals challenged with wild-type *Salmonella* Typhi (another enteric pathogen), who showed a decrease the percentage of CD8 T_EM_ cells expressing integrin α4β7 6–13 days after challenge [30] suggesting an early migration of these cells to the gut. Importantly, decrease in integrin α4β7+ cells was noted only in those individuals that developed disease after challenge. Homing of immune cells is a very dynamic process and in the current study, we did not collect earlier time points that have allowed a more in depth analysis of the migration process of these cells. Future studies will address this issue in more detail.

The current study describes the T-CMI responses to a vaccine candidate. Therefore, it is not possible to identify immunological correlates of protection. Future studies in which volunteers will be orally immunized and subsequently exposed to wild-type *Shigella flexneri* 2a hold the potential to identify defined T-CMI responses associated with protection from shigellosis. The novel technique presented in this manuscript will be of importance in this endeavor.

In sum, we have developed a novel method for measuring T-CMI to IpaB, which is an immunogenic protein present in all *Shigella* species and a component of some current subunit vaccine candidates [16, 17]. We used this technique to demonstrate the induction of T-CMI, and characterized in depth, for the first time, these responses, including the T memory subsets elicited and their homing potential, following exposure to CVD 1208S, a leading attenuated *S. flexneri* 2a vaccine candidate. Upcoming studies involving vaccination and/or challenge with attenuated and wild-type *S. flexneri* organisms will be essential in elucidating the role of T-CMI in controlling *Shigella* infection.

## Conclusions

In this manuscript we describe the development of a new method involving the use of IpaB-LPS-nanoparticles to measure T-CMI against IpaB, a major protein involved in *Shigella* pathogenesis. We demonstrate in humans immunized with CVD 1208S, a leading attenuated *S. flexneri* 2a vaccine candidate, the induction of CD4 and CD8 T-CMI responses as indicated by cytokine production by the different memory subsets following stimulation with B-LCL loaded with IpaB-LPS-nanoparticles. The cells that showed the strongest and most diverse responses belonged to the CD8 T_EM_ subset. In this population, after 3 doses of CVD 1208S, 72.2% of the vaccinated volunteers produced at least one cytokine. Furthermore, these cells showed multifunctional capabilities, especially those producing TNF-α. These results add further support for the continuing development of CVD 1208S as a vaccine against shigellosis.

## Additional file

**Additional file 1.** Additional figures and table.

## Abbreviations

MCB: Master Cell Bank
CFU: colony forming units
B-LCL: EBV-transformed B-lymphocytic cell lines
T3SS: type 3 secretion system
IFN-γ: interferon gamma
TNF-α: tumor necrosis factor alpha
IL-6: interleukin 6
IL-4: interleukin 4
T_EM_: T effector memory
T_EMRA_: T effector memory CD45-RA+
T_CM_: T central memory
BCR: B cell receptor
mAb: monoclonal antibody
TI-1: T cell independent antigen type 1
RIG-I: RNA sensor retinoic acid-inducible gene I
