# Supplementary material for: T cell mediated immunity induced by the live-attenuated Shigella flexneri 2a vaccine candidate CVD 1208S in humans
Source: J Transl Med. 2018 Mar 13;16:61. doi: 10.1186/s12967-018-1439-1 (PMC5851169; doi:10.1186/s12967-018-1439-1)
Supplement: Supplementary file 1 — Additional file 1. Additional figures and table. [file 12967_2018_1439_MOESM1_ESM.docx]

**
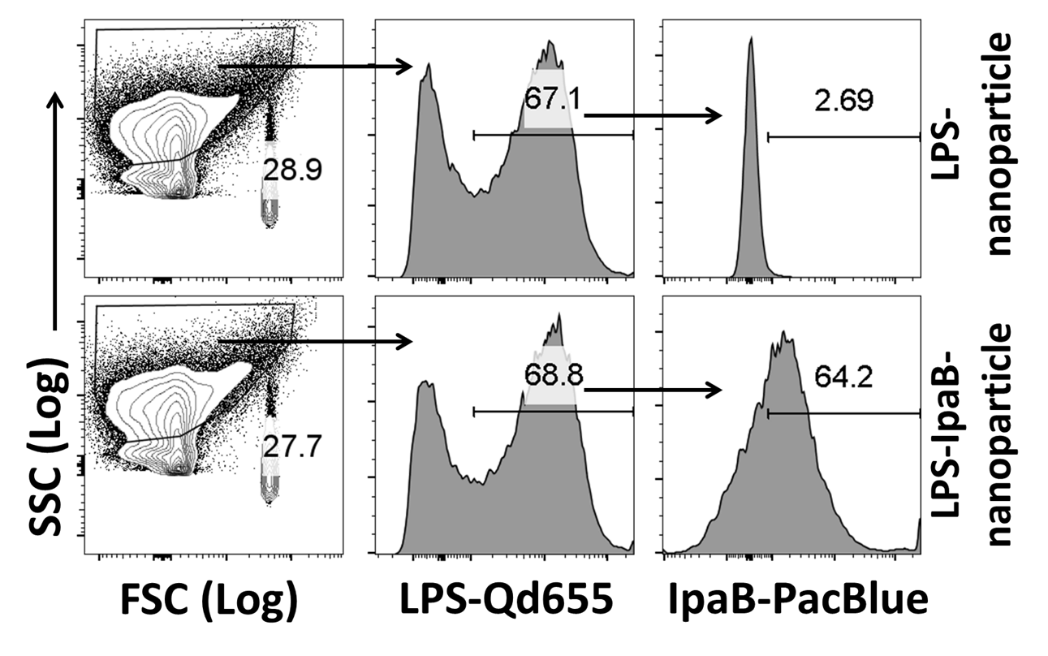
**

**Figure S1. Incorporation of IpaB into LPS-nanopaticles.** *S. flexneri* 2a lipopolysaccharide (LPS)-QDot655 micelles of nanoparticle size (approx. 30-60 nm) (LPS-nanoparticles) were generated as we had previously described [1-5] (Top Panels). *S. flexneri* IpaB labeled with Pacific Blue was incorporated into the LPS-nanoparticle order to generate LPS-IpaB-nanoparticles (Bottom Panels). See materials and methods for details. Incorporation of IpaB into the LPS-nanoparticles was evaluated by flow cytometry.


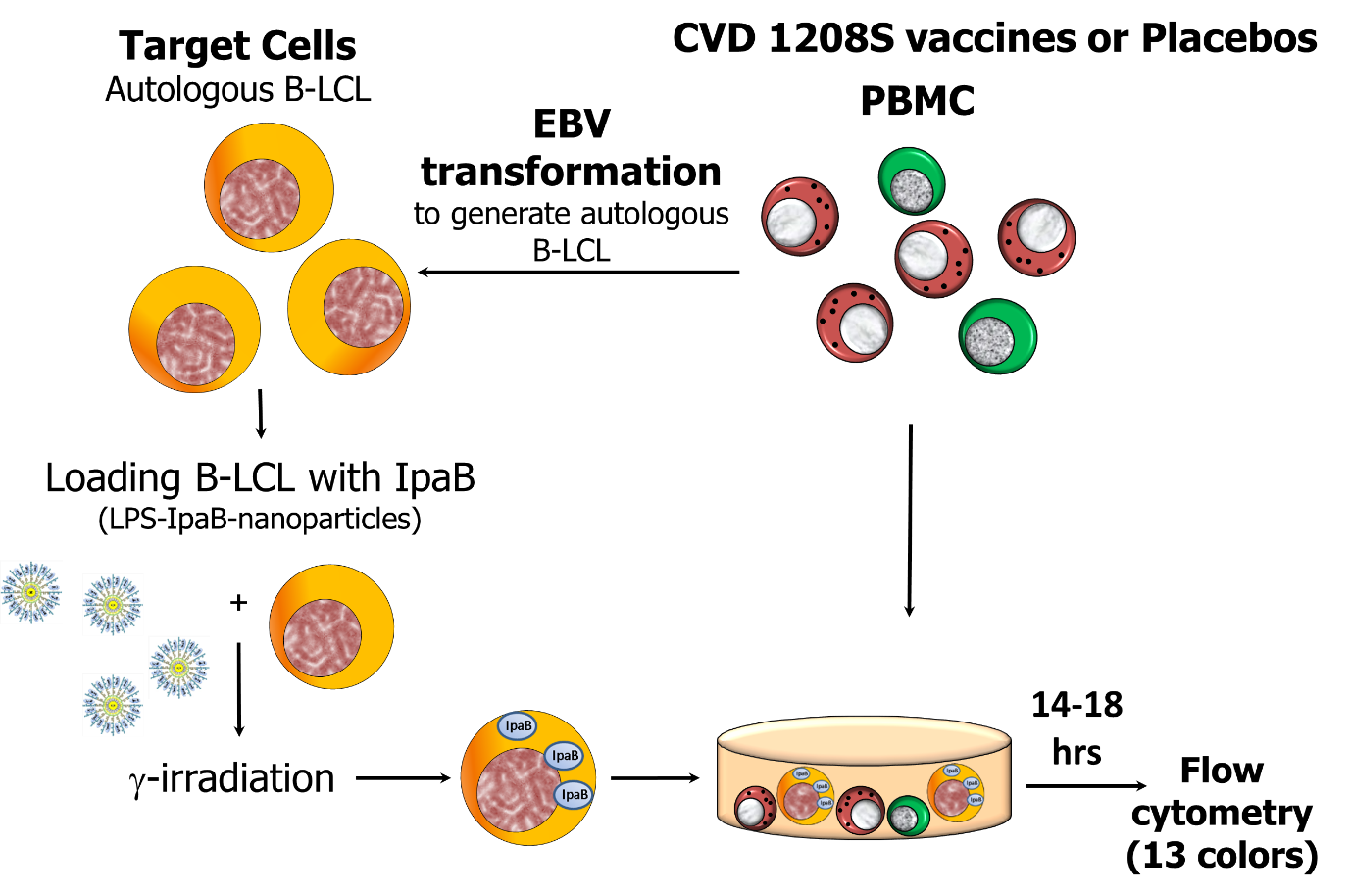


**Figure S2. Summary of experimental design to evaluate T-CMI to *Shigella* IpaB.** PBMC from vaccinated volunteers were transformed with EBV to generate autologous B-LCL. Subsequently, B-LCL were exposed to LPS-IpaB-nanopaticles for 16-18 hours. B-LCL were then gamma-irradiated and used as stimulator /target cells by exposing them to PBMC from CVD 1208S or placebo vaccinees (14-18 hours). Subsequently, induction of T-CMI (cytokine production and/or CD107a upregulation) immunity was analyzed in a LSRII flow cytometer.


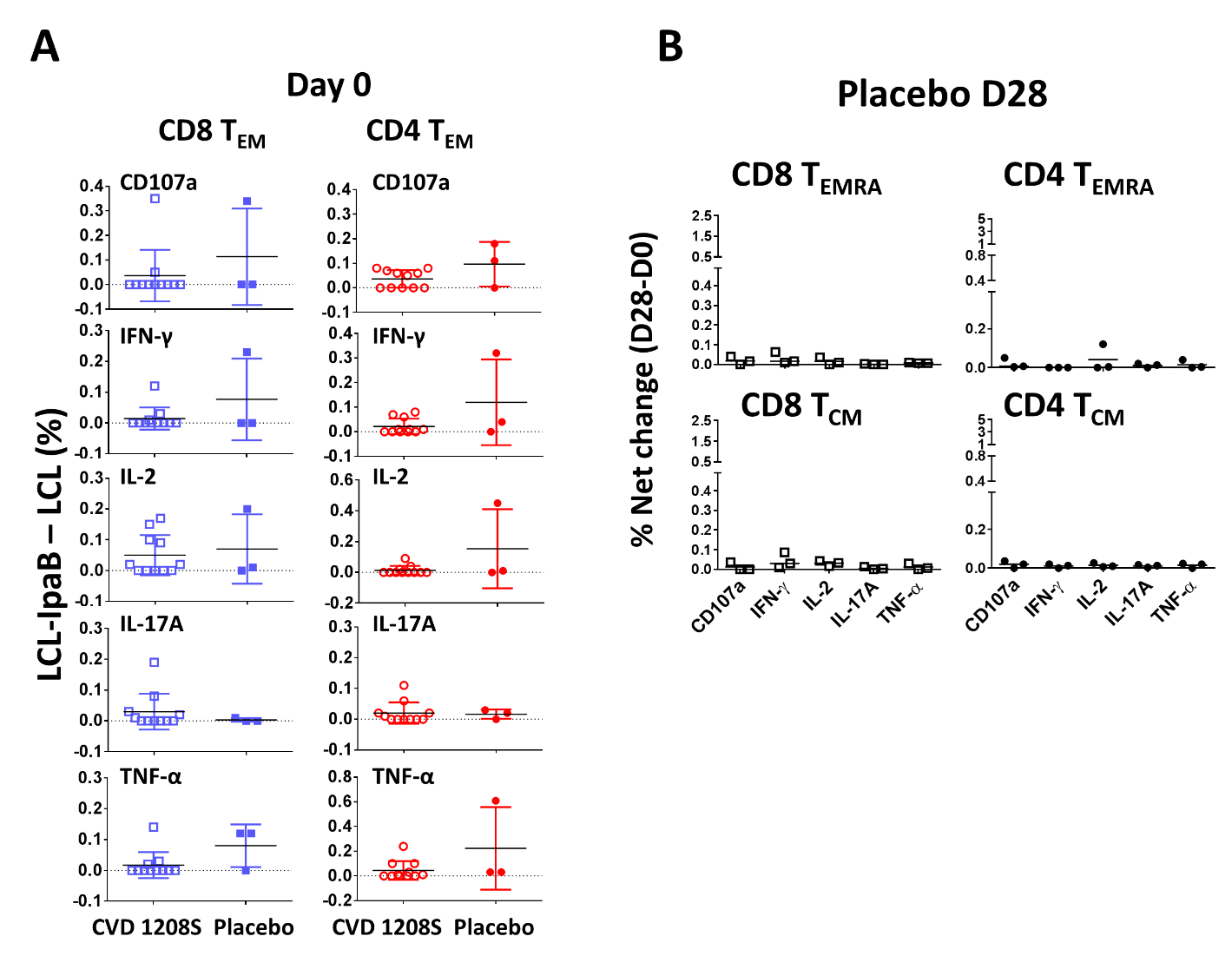


**Figure S3**. **A**. Cytokine production and CD107a expression at baseline (day 0) by CD8 and CD4 T_EM_ cells. No differences between CDV 1208S vaccinees and placebo recipients were noted before vaccination. **B**. Net percentage changes in cytokine or CD107a expression in CD8 T_EMRA_, CD4 T_EMRA_, CD8 T_CM_ and CD4 T_CM_ placebo recipients, compared to day 0, 28 days after the first vaccine dose. Minimal responses were identified among placebo recipients.

**Table S1. Vaccinees showing increased expression of cytokines or the degranulation marker CD107a.** The table shows the number and corresponding percentage (%) of volunteers that exhibited increased expression of cytokines (IFN-y, IL-2, IL-17A and TNF-α) or CD107a 28 days after each immunization. The data are displayed for the various CD8 and CD4 memory subsets.

**Additional information references**

1. Betanzos CM, Gonzalez-Moa M, Johnston SA, Svarovsky SA: **Facile labeling of lipoglycans with quantum dots.** *Biochemical and Biophysical Research Communications* 2009, **380:**1-4.

2. Anderson RE, Chan WCW: **Systematic Investigation of Preparing Biocompatible, Single, and Small ZnS-Capped CdSe Quantum Dots with Amphiphilic Polymers.** *ACS Nano* 2008, **2:**1341-1352.

3. Dubertret B, Skourides P, Norris DJ, Noireaux V, Brivanlou AH, Libchaber A: **In Vivo Imaging of Quantum Dots Encapsulated in Phospholipid Micelles.** *Science* 2002, **298:**1759-1762.

4. Toapanta FR, Bernal PJ, Fresnay S, Darton TC, Jones C, Waddington CS, Blohmke CJ, Dougan G, Angus B, Levine MM, et al: **Oral Wild-Type Salmonella Typhi Challenge Induces Activation of Circulating Monocytes and Dendritic Cells in Individuals Who Develop Typhoid Disease.** *PLoS Negl Trop Dis* 2015, **9:**e0003837.

5. Toapanta FR, Bernal PJ, Fresnay S, Magder LS, Darton TC, Jones C, Waddington CS, Blohmke CJ, Angus B, Levine MM, et al: **Oral Challenge with Wild-Type Salmonella Typhi Induces Distinct Changes in B Cell Subsets in Individuals Who Develop Typhoid Disease.** *PLoS Negl Trop Dis* 2016, **10:**e0004766.
